# Clinical and treatment profiles of arterial hypertension in Mexico during the COVID-19 pandemic: a cross-sectional survey endorsed by the “Collaborative Group on Arterial Hypertension”

**DOI:** 10.3389/fpubh.2024.1385349

**Published:** 2024-07-12

**Authors:** Silvia Palomo-Piñón, Neftali Eduardo Antonio-Villa, Ricardo Alfonso Rangel-Zertuche, María Guadalupe Berumen-Lechuga, Julio Manuel Medina-Serrano, Luis Rey García-Cortés, Oliva Mejia-Rodríguez, María de la Luz León-Vázquez, Roxana del Socorro González-Dzib, Vidal José González-Coronado, Cleto Álvarez-Aguilar, José Ramón Paniagua-Sierra, Luis Alcocer, the Collaborative Group on Arterial Hypertension from the Mexican Institute of Social Security

**Affiliations:** ^1^Unidad de Investigación Médica en Enfermedades Nefrológicas Siglo XXI (UIMENSXII), Unidad Médica de Alta Especialidad Hospital de Especialidades “Dr. Bernardo Sepúlveda G” Centro Médico Nacional Siglo XXI, Instituto Mexicano del Seguro Social, Mexico City, Mexico; ^2^Grupo de Expertos en Hipertensión Arterial México (GREHTA), Mexico City, Mexico; ^3^Grupo Colaborativo en Hipertensión Arterial (GCHTA), Mexico City, Mexico; ^4^Departamento de Endocrinología, Instituto Nacional de Cardiología Ignacio Chávez, Mexico City, Mexico; ^5^Jefatura Prestaciones Médicas, Órgano de Operación Administrativa Desconcentrada, Instituto Mexicano del Seguro Social, Coahuila, Mexico; ^6^Jefatura de Servicios de Prestaciones Médicas, Órgano de Operación Administrativa Desconcentrada México Poniente, Toluca, Mexico; ^7^Coordinación de Planeación y Enlace Institucional, Órgano de Operación Administrativa Desconcentrada en Sinaloa, Instituto Mexicano del Seguro Social, Culiacán, Sinaloa, Mexico; ^8^Coordinación de Planeación y Enlace Institucional, Jefatura de Servicios de Prestaciones Médicas, Órgano de Operación Administrativa Desconcentrada Regional Estado de México Oriente, Instituto Mexicano del Seguro Social, Oriente, Mexico City, Mexico; ^9^Centro de Investigación Biomédica de Michoacán del Instituto Mexicano del Seguro Social and by Escuela de Medicina, Campus Morelia, UVAQ, Michoacán, Mexico; ^10^Coordinación de Planeación e Enlace Institucional, Órgano Operativo de Administración Desconcentrada en Tlaxcala, Instituto Mexicano del Seguro Social, Tlaxcala, Mexico; ^11^Coordinación de Investigación en Salud, Instituto Mexicano del Seguro Social en Campeche, Campeche, Mexico; ^12^Departamento de Cardiología Hospital Regional “1 Octubre”, Instituto de Seguridad y Servicios Sociales para los Trabajadores del Estado, Mexico City, Mexico; ^13^Facultad de Ciencias Médicas y Biológicas “Dr. Ignacio Chávez”, Universidad Michoacana de San Nicolás de Hidalgo, Morelia, Michoacan, Mexico; ^14^Jefe de la Unidad de Investigación Médica en Enfermedades Nefrológicas Siglo XXI, Unidad Médica de Alta Especialidad Hospital de Especialidades “Dr. Bernardo Sepúlveda G” - Centro Médico Nacional Siglo XXI, Instituto Mexicano del Seguro Social, Mexico City, Mexico; ^15^Instituto Mexicano de Salud Cardiovascular, Mexico City, Mexico

**Keywords:** hypertension, blood pressure, epidemiology, public health, disease management, COVID-19

## Abstract

**Background:**

Arterial hypertension is highly prevalent in Mexico; nevertheless, there are limited insights regarding its management during the COVID-19 pandemic. Here, we estimate the prevalence of clinical and treatment profiles of arterial hypertension and explore associated factors for undiagnosed and uncontrolled hypertension using a cross-sectional survey endorsed by the Collaborative Group on Arterial Hypertension from the Mexican Institute of Social Security.

**Methods:**

Our survey was conducted from May to November 2021 using the May-Measurement Month 2021 protocols of the International Society of Hypertension. Arterial hypertension (defined as: blood pressure [BP] ≥140/90 mmHg, previous diagnosis, or taking antihypertensives) and its clinical and treatment profiles were classified according to the World Hypertension League Expert Committee. Mixed-effects logistic regression models were used to explore associated factors for undiagnosed and uncontrolled hypertension.

**Results:**

Among 77,145 screened participants (women: 62.4%; median age: 46 [IQR: 32–59] years), the prevalence of arterial hypertension was 35.7% (95% CI: 35.3–36.0, *n* = 27,540). Among participants with arterial hypertension, 30.9% (95% CI: 30.4–31.5, *n* = 8,533) were undiagnosed, 6.6% (95% CI: 6.3%−6.9%, *n* = 1,806) were diagnosed but untreated, 43.4% (95% CI: 42.9–44.0, *n* = 11,965) had uncontrolled hypertension, and only 19% (95% CI: 18.6%−19.5%, *n* = 5,236) achieved hypertension control (BP < 130/80 mmHg). Explored associated factors for undiagnosed and uncontrolled hypertension include being men, living in the central and southern regions, lower educational attainments, higher use of pharmacological agents, and previous COVID-19 infection.

**Conclusion:**

Our findings suggest that adverse arterial hypertension profiles, mainly undiagnosed and uncontrolled hypertension, were highly prevalent during the context of the COVID-19 pandemic in Mexico.

## Introduction

Arterial hypertension is a central contributor to the burden of chronic health diseases worldwide (1). The high prevalence of arterial hypertension reported within low- and middle-income countries (LMICs) has brought substantial consequences, as it has been linked directly responsible for over 1.6 million deaths annually within Latin America, mainly related to cardiovascular diseases (CVD) (2–4). The management of arterial hypertension represents a challenging situation for healthcare systems in LMICs, as there is a high proportion of unawareness and uncontrolled hypertension in the general population (5). Moreover, Latin America has historically suffered from underfunded healthcare systems that limit the coverage and access to adequate screening and sufficient antihypertensive treatment, particularly in primary-care settings (6).

Mexico has experienced a steep increase in arterial hypertension prevalence and mortality within the last two decades (7). Furthermore, the Mexican population coexists with a high prevalence of cardiometabolic diseases and risk factors that have demonstrated to impact the management of blood pressure (8, 9). These structural conditions created a challenging scenario for managing arterial hypertension within the context of the coronavirus disease 2019 (COVID-19) pandemic. It has been reported that the Mexican healthcare system modified its care policies to prioritize the attention of critically ill COVID-19 patients, triggering structural deficiencies in care in different healthcare sectors (10, 11). Consequently, these changes brought a deficiency in care for other chronic health diseases, such as diabetes and cardiovascular diseases (12, 13). We hypothesize that arterial hypertension was not the exception, as modification in healthcare policies could have led to an increase in the burden of undiagnosed and uncontrolled hypertension. Though several reports have estimated the impact of the COVID-19 pandemic on chronic health conditions and its related complications mainly related to excess mortality, there are limited insights regarding the clinical and treatment management of arterial hypertension during the COVID-19 pandemic in Mexico (12, 14). Hence, there is a need to assess the epidemiological situation of arterial hypertension profiles to strengthen healthcare policies and mitigate the burden of hypertension in our country.

Hence, this study aimed to (1) estimate the prevalence of clinical and treatment profiles of arterial hypertension and (2) explore associated factors for undiagnosed and uncontrolled hypertension during the COVID-19 pandemic using a cross-sectional survey using a cross-sectional survey endorsed by the Collaborative Group on Arterial Hypertension from the Mexican Institute of Social Security.

## Methods

### Study design

We performed a cross-sectional survey among adults ≥20 years living in Mexico between May to November 2021 following the international protocol established by the May-Measurement Month (MMM) 2021 consortium by the International Society of Hypertension (ISH) (15). In Mexico, reports of the MMM protocols have been published elsewhere (16, 17). Briefly, this survey consisted of an open invitation of adults to assist with modules disposed by our group of study in public healthcare clinics across Mexico. The participation consisted of a standardized arterial pressure measurement followed by a standardized questionnaire to ask for sociodemographic, clinical, and lifestyle habits and arterial hypertension treatment-related variables. This survey followed the recommendations of the 2021 version of MMM, including a section to interrogate COVID-19 related variables. All the modules involved trained healthcare personnel previously certified by qualified physicians to measure clinical and arterial blood pressure according to standardized guidelines. The Investigation Review Board of the Mexican Institute of Social Security (Acronym in Spanish—IMSS: Instituto Mexicano del Seguro Social) approved this study by protocol number R-2021-1406-016 (Supplementary Figure 1). All the participants gave verbal informed consent before being assessed in the study and were given an internal identification number to anonymize their personal information. This study adhered to the STROBE guidelines for reporting cross-sectional studies (Supplementary Table 1).

### Variables and definitions

#### Outcome variable

Our main analysis focused on assessing the clinical and treatment profiles of people with arterial hypertension. This classification is based on the definition of arterial hypertension adopted by the World Hypertension League Expert Committee (WHLEC) for epidemiological studies to ensure comparison across countries and consistency with other international surveys, facilitating the evaluation of the impact of public health policies across time (18).

*I. Arterial hypertension*—According to the WHLEC, a person is considered to have arterial hypertension if they meet any of the following criteria: (1) systolic and/or diastolic blood pressure readings >140/90 mmHg, (2) have previously been diagnosed with arterial hypertension, or (3) was taking any antihypertensive medication of drug to regulate their high blood pressure.
*II. Clinical and treatment profiles*—Undiagnosed arterial hypertension was considered when a participant had systolic and/or diastolic blood pressure readings greater than 140 and 90 mmHg, respectively, and was not aware of having any arterial hypertension diagnosis, or neither had any antihypertensive treatment. Untreated arterial hypertension was classified when a participant had a previous arterial hypertension diagnosis but was not receiving any antihypertensive treatment. Treated arterial hypertension was considered when a participant had a previous arterial hypertension diagnosis and self-reported to be receiving antihypertensive treatment. Uncontrolled arterial hypertension was defined as a participant who had been diagnosed with arterial hypertension and was treated with any antihypertensive treatment but whose blood pressure was greater or equal to 130 or 80 mmHg. Controlled arterial hypertension was considered when a diagnosed and treated individual had blood pressure lower than 130 or 80 mmHg.

#### Arterial blood pressure assessment

Arterial blood pressure was measured using a brand-name digital sphygmomanometer (*OMRON HEM-9200T*) available and provided for all medical facilities. Three measurements of blood pressure were taken, each with a one-minute break in between. The results of the final two readings were then averaged and used in all analyses to determine the participant's arterial blood pressure.

#### Standardized questionnaire assessment

A) *Sociodemographic variables—*We included age, sex, state of residency, years of education (categorized as 0–6, 7–12, and ≥13 years), and whether the participant self-identified as Mexican-Mestizo, Caucasian, or Afro-descendant as our sociodemographic variables. For convenience, participants were grouped as living in four regions in Mexico: north, central, metropolitan area, and south region based on the classification of the Mexican National Institute of Geography (Acronym in Spanish—INEGI: Instituto Nacional de Estadística, Geografía e Informática) (19).
B) *Clinical and lifestyle habits evaluation—*Clinical variables asked in the questionnaire were time categorized in 12 months since the last clinical visit to a healthcare professional, smoking and alcohol consumption, aspirin and statin use, and prior clinical diagnosis of diabetes, ischemic heart disease (IHD), or stroke. For the anthropometric evaluation, weight was measured in kilograms using calibrated scales. Self-reported weight was captured in participants in which weight could not be directly assessed. All participants received standardized dietary and lifestyle recommendations in an informative card (Supplementary Figure 1), and routine medical follow-up was advised.
C) *Hypertension-related variables—*A direct questionnaire was applied to all participants, asking whether a medical professional had previously informed them if they had been diagnosed with arterial hypertension by asking, “*Have you ever been informed by a doctor or other health professional that you had arterial hypertension, also known as high blood pressure?*”. The following query was also used to determine whether a person was taking antihypertensives: “*Are you now taking any drugs, tablets, or pills for high blood pressure?*”. If the answer to the previous question was positive, we asked the total number of medications using the following query: “*How many drugs, tablets, or pills are you currently taking for managing your hypertension?*”. For convenience, we classified the antihypertensive treatment as monotherapy, dual therapy, and triple therapy.
D) *COVID-19-related variables—*Participants were asked whether they had previous COVID-19 infection as the response to the following question: “*Have you had any positive test for COVID-19 (Coronavirus) disease?*”. Additionally, it was asked whether their hypertension treatment was affected by COVID-19 using the question: “*Was your arterial hypertension treatment affected due to the COVID-19 pandemic?*”. Finally, COVID-19 vaccination was asked through the query: “*Have you already received any COVID-19 vaccine?*”.

### Statistical analysis

Continuous data is presented in median and interquartile range [IQR]. Categorical variables are presented as frequency and in absolute proportion. All statistical analyses were performed in R Studio (Version 4.1.2). A value of p < 0.05 was considered as our statistically significance threshold.

#### Missing variables assessment

To calculate the missing values from continuous variables, we used a multiple imputation algorithm based on the fully conditional specification technique as proposed by Van Buuren and Groothuis-Oudshoorn under the assumption that data was missed completely at random. We multiply 5 imputed datasets for a maximum of 5 iterations combined using Rubin's rules using the *mice* package (Version 3.14.0) (20). Detailed results of imputed variables are presented in Supplementary Figure 2.

#### Prevalence estimation of clinical and treatment profiles of arterial hypertension

The Clopper-Pearson approach was used to estimate the overall prevalence of arterial hypertension, along with clinical and treatment hypertension profiles. We further stratify these prevalences across sex, region of residency, ethnicity, and educational attainments. We used the *epiR* package to estimate the prevalence with a 95% confidence interval (Version 2.0.3) (21). The *networkD3* (Version 0.4) package was used to create Sankey-Diagrams and bar plots to visualize the clinical and treatment profiles related to arterial hypertension stratified by sociodemographic variables (22).

#### Factors related to undiagnosed and uncontrolled arterial hypertension

To investigate the potential factors associated with undiagnosed and uncontrolled arterial hypertension, we fitted random-effects binomial logistic regression models to examine the roles of sociodemographic, clinical, lifestyle habits, arterial hypertension treatment, and COVID-19-related variables. The final models were chosen according to the lowest Bayesian Information Criteria (BIC). A model with multicollinearity in its estimation was judged to have a Variance Inflation Factor (VIF) >5. The *jtools* package (Version 2.1.4) was used to build odds-ratio charts (23).

#### Sensitivity analyses

The estimated prevalence of arterial hypertension depends on the definition criteria proposed by different statements and societies. As a sensitivity analysis to evaluate whether a lower arterial blood pressure threshold may modify the prevalence of clinical and treatment profiles of arterial hypertension, we tested the American Heart Association (AHA) definition (24). The AHA considers arterial hypertension when an individual has systolic and/or diastolic blood pressure readings >130 and 80 mmHg, respectively, the use of antihypertensives and previous medical diagnosis of hypertension.

## Results

### Study population

Throughout the study period, 77,145 participants were screened across 13 states in Mexico. The number of participants contributed by each state is displayed in Supplementary Table 2, and the complete descriptive characteristics of the overall study population are presented in Supplementary Table 3. Briefly, our sample predominantly consisted of women (62.4%), with a median age of 46 years (IQR: 32-59). A significant portion of the participants, 51.2%, had 7 to 12 years of educational attainment, and most lived in the northern region of Mexico (49.6%). As of October 2022, 20% of the sample had previously self-reported COVID-19 disease and 63.4% had received vaccinations against the SARS-CoV-2 virus.

### Prevalence of arterial hypertension in Mexico during the COVID-19 pandemic

**Figure 1:**
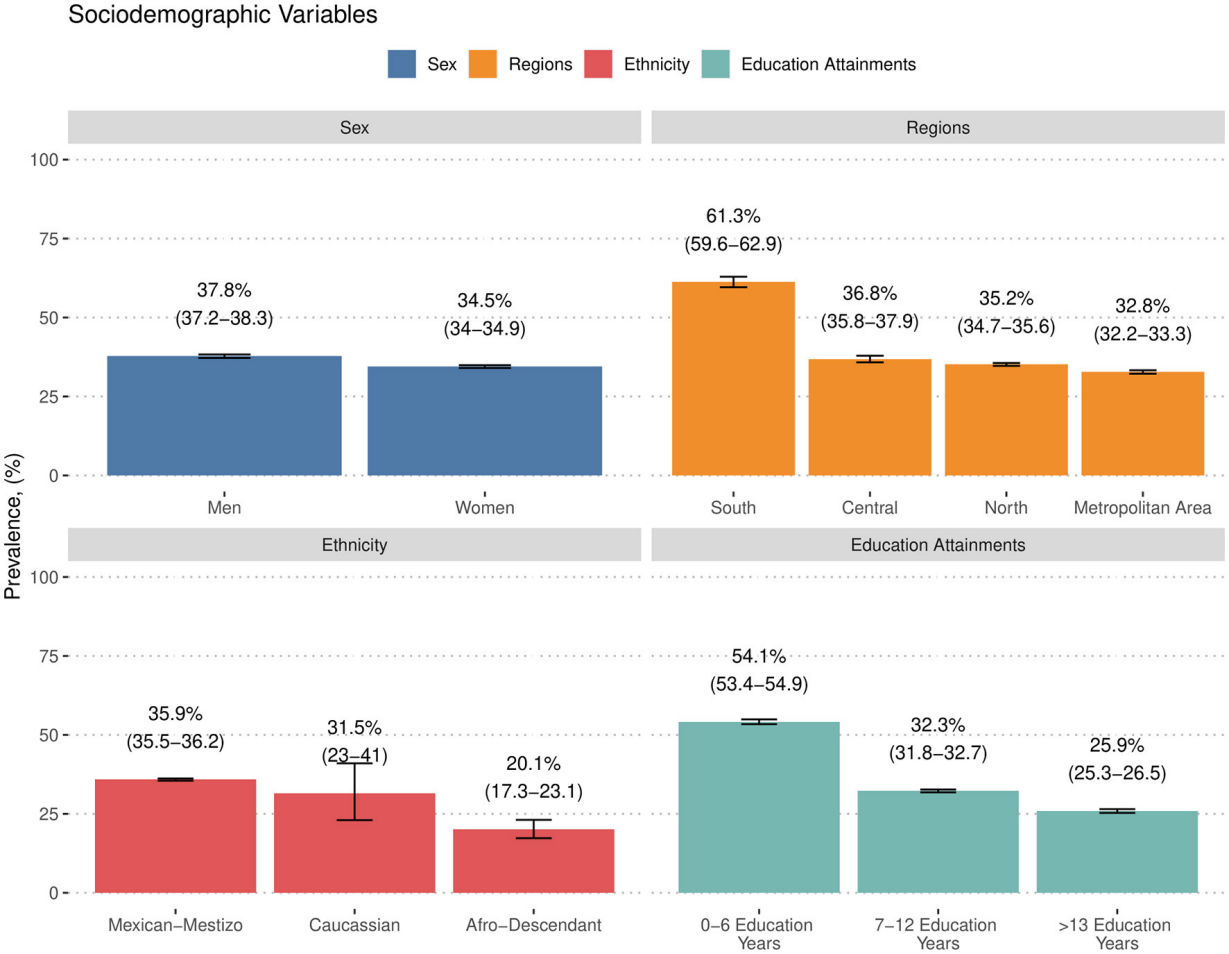
Prevalence of arterial hypertension stratified by sex, regions in Mexico, ethnicity, and educational attainments.

We identified 27,540 participants with arterial hypertension during the studied period, resulting in an estimated prevalence of 35.7% (95% CI: 35.3% to 36.0%). The characteristics of these participants, stratified by clinical and treatment profiles, are detailed in Table 1. Sociodemographic stratification of arterial hypertension indicated a higher prevalence among male participants (37.8%, 95% CI: 37.2% to 38.3%), participants living in the southern region (61.3%, 95% CI: 59.6% to 62.9%), those of Mexican-Mestizo ethnicity (35.9%, 95% CI: 35.5% to 36.2%), and particularly among those with 0 to 6 years of educational attainment (54.1%, 95% CI: 53.4% to 54.9%) (Figure 1).

**Table 1:** Descriptive characteristics of the population living with arterial hypertension identified in Mexico.

**Characteristic**	**Participants with hypertension (*n* = 27,540)**	**Undiagnosed (*n* = 8,533)**	**Untreated (*n* = 1,806)**	**Uncontrolled (*n* = 11,965)**	**Controlled (*n* = 5,236)**
Women, (%)	60.3%	54.1%	64.8%	60.5%	68.1%
Men, (%)	39.7%	45.9%	35.2%	39.5%	31.9%
Age, (Years) [median, IQR]	56 (45, 66)	51 (39, 62)	46 (35, 58)	59 (50, 67)	59 (49, 67)
0-6 Years of Education, (%)	35.1%	27.8%	25.1%	39.5%	40.5%
7-12 Years of Education, (%)	46.2%	49.5%	49.4%	45.0%	42.6%
>13 Years of Education, (%)	18.7%	22.8%	25.4%	15.5%	16.8%
North Region, (%)	48.8%	54.1%	34.3%	49.9%	42.9%
Central Region, (%)	10.9%	17.3%	15.7%	7.8%	5.9%
Metropolitan Area, (%)	32.7%	22.4%	45.2%	33.8%	42.8%
South Region, (%)	7.6%	6.2%	4.8%	8.6%	8.4%
Caucasian Ethnicity, (%)	0.1%	0.1%	0.3%	0.1%	0.2%
Mexican–Mestizo Ethnicity, (%)	99.3%	98.7%	99.0%	99.7%	99.6%
Afro–Descendant Ethnicity, (%)	0.5%	1.2%	0.7%	0.2%	0.2%
Performs Any Physical Activity, (%)	30.4%	32.3%	32.3%	28.4%	31.3%
Never-Smoking, (%)	60.4%	63.6%	57.2%	58.1%	61.4%
Quit-Smoking, (%)	25.5%	19.6%	24.5%	29.4%	26.7%
Active-Smoking, (%)	14.1%	16.8%	18.3%	12.5%	11.9%
Never-Drink Alcohol, (%)	77.3%	72.6%	71.0%	78.7%	83.9%
Frequent Intake Alcohol, (%)	17.8%	21.3%	22.8%	16.7%	12.9%
Daily Intake Alcohol, (%)	4.9%	6.1%	6.2%	4.7%	3.2%
High Arterial Blood Pressure During Pregnancy, (%)*	11.5%	6.3%	27.7%	12.1%	11.7%
Current Pregnancy, (%)*	2.3%	2.9%	7.9%	1.5%	1.5%
Diabetes, (%)	26.8%	11.1%	19.5%	35.8%	34.5%
Previous CVD, (%)	6.0%	2.8%	4.5%	7.9%	7.3%
Previous Hearth Attack, (%)	4.6%	2.1%	2.7%	6.1%	5.8%
Previous Stroke, (%)	1.8%	0.9%	2.0%	2.5%	1.8%
Statin Use, (%)	15.6%	6.2%	7.0%	21.1%	21.2%
Aspirin Use, (%)	19.3%	7.7%	15.2%	25.6%	25.6%
Previous COVID-19 Infection, (%)	20.1%	22.9%	23.9%	18.5%	18.0%
Antihypertensive Treatment Affected by COVID-19, (%)	8.3%	5.6%	5.8%	11.0%	7.3%
COVID-19 Vaccine, (%)	75.5%	67.6%	63.7%	80.3%	81.7%
<12 Months Since Clinical Visit, (%)	83.7%	71.6%	71.8%	90.5%	92.0%
≥12 Months Since Clinical Visit, (%)	12.9%	22.7%	23.1%	7.3%	5.9%
Never had a Clinical Visit, (%)	3.4%	5.7%	5.1%	2.2%	2.1%
No-Therapy, (%)	37.5%	100.0%	100.0%	0.0%	0.0%
Monotherapy Antihypertensive Treatment, (%)	33.8%	0.0%	0.0%	52.7%	57.6%
Dual-Therapy Antihypertensive Treatment, (%)	21.5%	0.0%	0.0%	35.4%	32.4%
Triple-Therapy Antihypertensive Treatment, (%)	7.1%	0.0%	0.0%	12.0%	10.0%
Weight, (kg) [median, IQR]	75 (66, 86)	76 (67, 87)	73 (64, 85)	76 (67, 87)	72 (63, 81)
SBP, (mmHg) [median, IQR]	133 (121, 144)	141 (130, 149)	122 (112, 133)	137 (130, 147)	116 (109, 123)
DBP, (mmHg) [median, IQR]	83 (76, 91)	91 (82, 94)	79 (72, 85)	85 (81, 91)	72 (68, 76)
HR, (bpm) [median, IQR]	76 (70, 84)	78 (71, 85)	77 (70, 84)	77 (70, 84)	74 (67, 81)

CVD, cardiovascular disease; mmHg, millimeters of mercury; bpm, beats per minute.

^*^Variables applicable only to women.

### Clinical and treatment profiles of arterial hypertension

**Figure 2:**
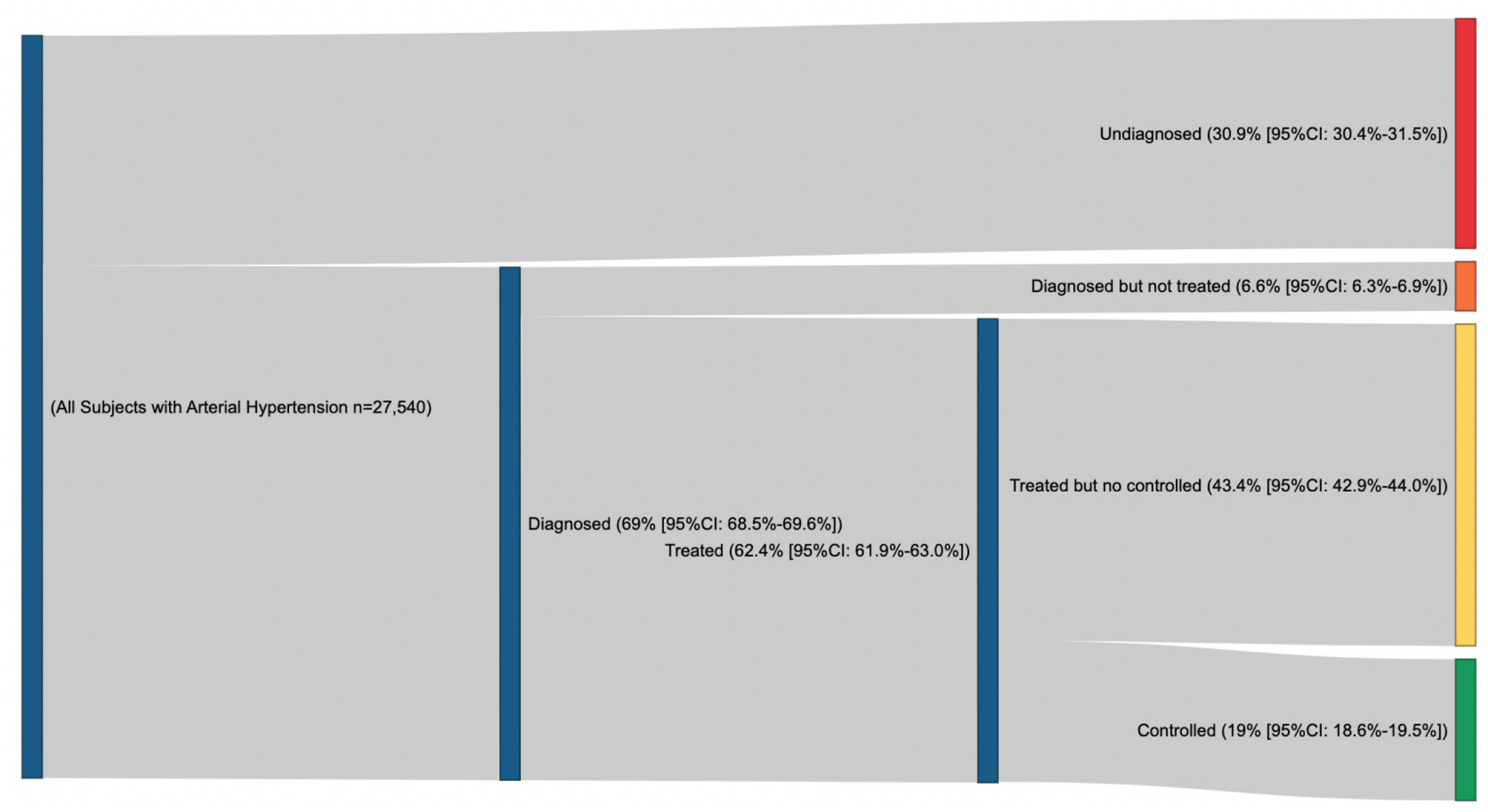
Proportion of undiagnosed, diagnosed but treated, treated but no controlled and controlled arterial hypertension.

Among all participants living with arterial hypertension (*n* = 27,540), we classified 30.9% (95% CI: 30.4% to 31.5%, *n* = 8,533) with undiagnosed hypertension and 6.6% (95% CI: 6.3% to 6.9%, *n* = 1,806) as previously diagnosed but currently untreated. The diagnosis and treatment of arterial hypertension were achieved in only 62.4% (95% CI: 61.9% to 63.0%, *n* = 17,201), of whom 43.4% (95% CI: 42.9% to 44.0%, *n* = 11,965) did not achieve arterial pressure goals and only 19% (95% CI: 18.6% to 19.5%, *n* = 5,236) were currently controlled (BP < 130/80 mmHg) (Figure 2).

### Prevalence of hypertension profile stratified by key-demographic variables

The stratification of clinical and treatment profiles by sociodemographic variables revealed that younger participants tended to have higher rates of undiagnosed and untreated arterial hypertension, while older participants were more likely to be on treatment but also exhibited a high prevalence of uncontrolled blood pressure. Notably, participants living in central states of Mexico, those identifying as Afro-descendant, and participants with higher educational levels yielded the highest prevalence of undiagnosed hypertension. Untreated hypertension was most prevalent among women, residents of the central region, Caucasian participants, and those with over 12 years of education. Additionally, the greatest prevalence of uncontrolled hypertension was observed in participants from the southern region with 0 to 6 years of educational attainment (Supplementary Table 4).

### Associated conditions for undiagnosed and uncontrolled arterial hypertension

The unadjusted regression model revealed several associated factors for undiagnosed and uncontrolled arterial hypertension (Supplementary Table 5). In the adjusted logistic regression analysis, we identify that being male, increased age, residing in the northern or southern regions, having lower educational levels, lack of self-reported physical activity, a history of COVID-19 infection, and usage of statins and aspirin emerged as significant associated factors of undiagnosed arterial hypertension compared to those with a known diagnosis. Additionally, being male, higher age, having 7–12 years of educational attainments, residing in the metropolitan, northern, or southern regions, frequent or daily alcohol consumption, not engaging in self-reported physical activity, previous stroke, reporting treatment disruption due to the COVID-19 pandemic and being on dual or triple antihypertensive therapy were identified as key contributing factors for uncontrolled hypertension compared with participants with controlled hypertension (Table 2).

**Table 2:** Adjusted binomial logistic regression model to evaluate the factors associated with undiagnosed and uncontrolled arterial hypertension in Mexico.

**Outcome**	**Variables**	**aOR**	**95% CI**	**p-value**
Undiagnosed hypertension (vs. no-hypertension) χ^2^(14) = 4,290.11, p < 0.01 Pseudo-R^2^ (McFadden) = 0.09 BIC: 44,369.42	**Sex, (%)**			<0.001
	Women	Ref	—	
	Men	1.47	1.40, 1.54	
	**Age categories, (%)**			<0.001
	18–35	Ref	—	
	36–49	1.54	1.39, 1.71	
	50–64	1.72	1.47, 2.02	
	>65	1.53	1.20, 1.94	
	**Education years, (%)**			<0.001
	>13	Ref	—	
	7–12	1.01	0.96, 1.08	
	0–6	1.23	1.15, 1.33	
	**Region of recruitment, (%)**			<0.001
	Central	Ref	—	
	Metropolitan area	2.08	1.96, 2.21	
	North	2.74	2.53, 2.96	
	South	3.89	3.45, 4.38	
	**Performing physical activity, (%)**			<0.001
	Yes	Ref	—	
	No	1.12	1.07, 1.18	
	**Previous COVID-19 infection, (%)**			<0.001
	No	Ref	—	
	Yes	1.22	1.15, 1.29	
	**Statin use, (%)**			<0.001
	No	Ref	—	
	Yes	1.69	1.50, 1.90	
	**Aspirin use, (%)**			<0.001
	No	Ref	—	
	Yes	1.90	1.71, 2.11	
Uncontrolled hypertension (vs. controlled) χ^2^(16) = 405.10, p < 0.01 Pseudo-R^2^ (McFadden) = 0.02 BIC = 20,902.35	**Sex, (%)**			**<0.001**
	Women	—	—	
	Men	1.34	1.25, 1.44	
	**Age categories, (%)**			**<0.001**
	18–35	Ref	—	
	36–49	1.46	1.24, 1.72	
	50–64	1.61	1.37, 1.88	
	>65	1.38	1.17, 1.62	
	**Education years, (%)**			**0.014**
	>13	Ref	—	
	7–12	1.15	1.05, 1.27	
	0–6	1.09	0.99, 1.21	
	**Region of recruitment, (%)**			**<0.001**
	Central	Ref	—	
	Metropolitan area	1.50	1.39, 1.61	
	North	1.40	1.21, 1.63	
	South	1.21	1.07, 1.38	
	**Alcohol intake, (%)**			**<0.001**
	Never-drink	Ref	—	
	Frequent intake	1.24	1.12, 1.37	
	Daily intake	1.32	1.10, 1.58	
	**Performing physical activity, (%)**			**<0.001**
	Yes	Ref	—	
	No	1.19	1.11, 1.28	
	**Previous stroke, (%)**			**0.047**
	No	Ref	—	
	Yes	1.27	1.00, 1.61	
	**Antihypertensive treatment affected by COVID-19, (%)**			**<0.001**
	No	Ref	—	
	Yes	1.52	1.34, 1.73	
	**Antihypertensive treatment, (%)**			**<0.001**
	Monotherapy	Ref	—	
	Dual-therapy	1.15	1.07, 1.24	
	Triple-therapy	1.23	1.11, 1.38	

aOR, Adjusted odds ratio; BIC, Bayesian information criteria. The bold value represent the name of the variable.

### Sensitivity analyses

Some reports in Mexico used the AHA definition for the classification of arterial hypertension (≤130/80 mmHg). Hence, we performed a sensitivity analysis to evaluate the prevalence of arterial hypertension using a blood pressure threshold <130/80 mmHg. We observed that the prevalence of arterial hypertension increased to 57.5% (95% CI: 57.1 to 57.8, *n* = 43,638) in the overall sample. The evaluation of the clinical and treatment profiles of arterial hypertension revealed that lowering the arterial blood pressure threshold increases the proportion of undiagnosed (56.4%, 95% CI: 55.9 to 56.9, *n* = 24,631) hypertension, but decreases the proportion of diagnosed (43.6%, 95% CI: 43.1 to 44.0, *n* = 19,007) and treated (39.4%, 95% CI: 38.9 to 39.9, *n* = 17,201) arterial hypertension. Hence, a lower proportion of uncontrolled (27.4%, 95% CI: 27.0 to 27.8, *n* = 5,236) and controlled (11.9%, 95% CI: 11.7 to 12.3, *n* = 5,236) blood pressure was observed.

## Discussion

In this study, we aimed to determine the prevalence of clinical and treatment profiles of arterial hypertension as well as the associated factors for undiagnosed and uncontrolled hypertension, during the context of the COVID-19 pandemic in Mexico. We performed a cross-sectional survey of 77,145 participants and found that more than one-third of our sample was living with arterial hypertension, with nearly one-third of these cases undiagnosed, two-fifths uncontrolled, and only a fifth effectively reaching blood pressure goals. The stratification of clinical profiles underscored variability across different age groups and sociodemographic factors. Notably, being male, residing in the southern regions of Mexico, having lower educational levels, not performing physical activity, extensive use of pharmacological agents, and a history of COVID-19 infection were significantly associated with increased odds of both undiagnosed and uncontrolled hypertension. These findings underscore the magnitude of arterial hypertension as a critical public health concern during the COVID-19 pandemic, adding to the national burden of chronic diseases among adults in Mexico.

These results demonstrate a higher prevalence compared with previous studies performed by the MMM in Mexico (16, 17). Furthermore, a study performed by our group in the eastern zone of Mexico revealed a prevalence of 32.4% (95% CI: 31.2%-33.6%) within the studied sample, which suggests a higher prevalence at a national-wide level compared with previous estimations using the WHLEC definition (25). While our results denote an increased trend in prevalence of arterial hypertension over the past years, it is important to note that we are implementing a higher threshold compared with other studies. According to the National Health and Nutrition Survey (ENSANUT) conducted in 2018, 2020, and 2022, it was reported that 49.2%, 49.4%, and 47.8%, respectively, of the Mexican population were living with arterial hypertension using the AHA definition (>130/80 mmHg) (26–28). Our sensitivity analyses revealed a prevalence of 57.5%, which is higher compared to ENSANUT estimates. Our findings indicate that, even when considering a lower threshold, our results support the view that Mexico experienced an elevated prevalence of arterial hypertension during the COVID-19 pandemic. Nevertheless, our findings reinforce the need for a standardized consensus that contrasts the benefits of applying different definitions of arterial hypertension in Mexico in epidemiological studies and within prospective cohorts.

The potential explanations for the high prevalence of arterial hypertension in Mexico are linked with individual and sociodemographic components. Although arterial hypertension has been classified as a multifactorial disease, it has been identified that nutritional, behavioral, and environmental causes were combined with adverse sociodemographic conditions that directly impact the clinical and treatment presentations of arterial hypertension (1, 9). A possible explanation is that in Latin America, it has been reported that a high toll of socially disadvantaged populations experienced worse access to hypertension care during the last two decades, which led to an extensive challenge for managing chronic health diseases at primary care levels (4). Here, we demonstrated that participants within the central and southern regions experienced the highest prevalence of arterial hypertension compared with the rest of the country, driven by a high proportion of uncontrolled hypertensive disease. Similar results have been previously reported by a longitudinal study, which demonstrated that rural dwellers, uninsured subjects, and the least wealthy are at risk for persistent untreated and uncontrolled hypertension (29). In our results, factors that also contributed to both undiagnosed and uncontrolled disease include unhealthy lifestyle habits such as alcohol intake and lack of physical activity, which overall demonstrate that there is a need for targeting both individual and sociodemographic conditions to diminish the burden of arterial hypertension in Mexico.

During the context of the COVID-19 pandemic, Mexico experienced an interruption of primary healthcare services that mainly affected patients living with chronic health conditions. A time-series analysis performed in Mexico reported that over one-third of hypertensive care visits were delayed or postponed, and the proportion of controlled hypertensive disease declined by 17% (10). Though the disruptions were mainly driven by the hospital reconversion policy that sought to prioritize critically ill COVID-19 patients, the Mexican healthcare system was strained prior to the arrival of the pandemic (30). The structural deficiencies have been widely reported to be mainly characterized by a lack of healthcare personnel, inequalities in coverage and insufficient supplies for the primary care sector (31, 32). The COVID-19 pandemic exacerbated these deficiencies, leading to a proportion of the population living with chronic health conditions being exposed to acute complications and excess deaths due to diabetes and CVD, particularly within vulnerable groups (12, 13). Overall, the combination of structural factors and deficiencies in healthcare systems led to an increase of arterial hypertension in Mexico, particularly of undiagnosed and uncontrolled arterial hypertension in Mexico, consequently contributing to a high toll of deaths related to arterial hypertension reported during the COVID-19 pandemic.

Our results provide one of the first epidemiological estimations of the prevalence and treatment profiles of arterial hypertension in Mexico during the COVID-19 pandemic. We confirm that arterial hypertension remains a highly prevalent condition and a significant public health concern in Mexico. Future studies are needed to evaluate the impact of the normalization of health services and the strategies implemented to mitigate the pandemic, such as the intensive recruitment of medical personnel. However, there are still significant challenges in reducing the burden of this disease, which will require actions to promote healthier lifestyle habits, ensure primary care access for vulnerable populations, and provide adequate access to novel pharmacological therapies in order to reduce the high prevalence of arterial hypertension in Mexico.

### Strengths and limitations

Our study has strengths and limitations to be acknowledged. Among the strengths, we highlight the recruitment of 77,145 individuals, deriving in one of the largest samples performed in Mexico to estimate the prevalence of arterial hypertension. This estimation allowed us to study the prevalence of clinical and treatment profiles by regional, educational, and ethnic groups. Furthermore, we offer insights regarding associated factors for undiagnosed and uncontrolled arterial hypertension, which could be used to identify vulnerable groups within clinical practice. Nevertheless, some limitations need to be acknowledged. First, this survey was intended to be an open invitation to the general population to assist with provisional modules located in public clinics across Mexico. This could lead to a sampling bias toward capturing people who assisted with healthcare services for various reasons. Furthermore, due to COVID-19 mobility restrictions, we could only install modules in 13 of the 32 states of Mexico, leading to a potential underrepresentation compared with other national-wide probabilistic household surveys such as the ENSANUT. Second, we identify missing values regarding our arterial blood pressure assessment, a frequent issue reported by other studies worldwide. However, we used a multiple imputation algorithm approach to complete missing blood pressure values, which have demonstrated to derive unbiased estimations in previous studies and within our results. Third, although we explored and found associated factors for undiagnosed and untreated arterial hypertension, this survey is a cross-sectional design that could not make any causal association for the studied outcomes. Hence, future prospective studies should evaluate the impact of the observed associated factors in the development of acute and chronic complications related to undiagnosed and untreated arterial hypertension. Fourth, we were unable to assess height as a standardized measurement due to methodological and structural issues. Hence, we were unable to estimate body mass indexes in our study. Finally, we were unable to assess biochemical measurements and specific pharmacological treatments, which limited our capacity to provide details regarding therapeutic profiles, leading to future areas of opportunity and research.

## Conclusion

In conclusion, over one-third of our studied sample had arterial hypertension, in which one-third were classified with undiagnosed disease, two-fifths with uncontrolled blood pressure, and only one-fifth achieved controlled blood pressure. Key factors associated with these conditions included male sex, residing in the northern, southern, or central regions, lower educational attainment, physical inactivity, alcohol use, previous COVID-19 infection, and antihypertensive treatment disruptions due to the pandemic. These findings yield an urgent call to action to improve healthcare screening in primary care settings and guarantee sufficient arterial hypertension treatment to reduce the burden of the disease in Mexico during the ongoing COVID-19 pandemic and beyond.

## Data availability statement

The datasets presented in this article are not readily available because they contain sensitive information. Requests to access the datasets should be directed to SP-P at silvia-palomo@hotmail.com.

## Ethics statement

The studies involving humans were approved by the Investigation Review Board of the Mexican Institute of Social Security (Acronym in Spanish – IMSS: Instituto Mexicano del Seguro Social) by protocol number R-2021-1406-016. The studies were conducted in accordance with the local legislation and institutional requirements. The participants provided their written informed consent to participate in this study.

## Author contributions

SP-P: Conceptualization, Data curation, Funding acquisition, Investigation, Methodology, Resources, Supervision, Writing – original draft, Writing – review & editing. NA-V: Data curation, Formal analysis, Investigation, Methodology, Visualization, Writing – original draft, Writing – review & editing. RR-Z: Conceptualization, Data curation, Investigation, Validation, Writing – review & editing. MB-L: Conceptualization, Data curation, Investigation, Writing – review & editing. JM-S: Conceptualization, Data curation, Investigation, Writing – review & editing. LG-C: Conceptualization, Data curation, Investigation, Writing – review & editing. OM-R: Conceptualization, Data curation, Investigation, Writing – review & editing. ML-V: Conceptualization, Data curation, Investigation, Writing – review & editing. RG-D: Conceptualization, Data curation, Investigation, Writing – review & editing. VG-C: Conceptualization, Data curation, Investigation, Writing – review & editing. CÁ-A: Conceptualization, Data curation, Investigation, Writing – review & editing. JP-S: Conceptualization, Data curation, Investigation, Writing – review & editing. LA: Conceptualization, Data curation, Investigation, Writing – review & editing.
